# Investigation Into the Metabolism and Elimination Behavior of RU 58841, and Its Influence on the Steroid Profile in Humans

**DOI:** 10.1002/bmc.70602

**Published:** 2026-08-19

**Authors:** Aylin Okutan, Oliver Krug, Gregor Fußhöller, Thomas Piper, Mario Thevis

**Affiliations:** ^1^ Center for Preventive Doping Research, Institute of Biochemistry German Sport University Cologne Cologne Germany; ^2^ European Monitoring Center for Emerging Doping Agents Cologne/Bonn Germany

**Keywords:** anabolics, doping, masking agent, sport

## Abstract

RU 58841 is a nonsteroidal antiandrogen, developed for the treatment of androgen‐dependent conditions. This study aimed to characterize the human metabolism of RU 58841 for the first time and to assess its systemic exposure following topical administration. In an initial in vitro metabolism study, six potential metabolites were identified using liquid chromatography–high resolution (tandem) mass spectrometry (LC‐HRMS/MS). Subsequently, an in vivo administration study was conducted with a RU 58841‐containing hair treatment product, applied once to the scalp of six healthy male volunteers. Dried blood spots (DBS) and urine samples were collected and analyzed by LC‐low resolution MS/MS. Five in vivo‐derived metabolites of RU 58841 were detected in human urine, including products formed via oxidation, N‐dealkylation, and glucuronidation of the parent compound and its phase I metabolites. In DBS, the parent compound and the oxidized metabolite were detected, indicating considerable systemic exposure to topically applied RU 58841. The steroid profile (SP) as part of the Athlete Biological Passport (ABP) was not affected by the administration. These findings provide information about the metabolism and the systemic exposure of RU 58841, and further indicate that a single topical dose of 30 mg does not interfere with SP parameter determinations in routine doping controls.

## 1 Introduction

The pathophysiology of androgen‐related conditions such as androgenic alopecia (AGA) are primarily mediated by the binding of androgens to the androgen receptor (AR). The resulting conformational change into the AR's active form enables receptor dimerization, nuclear translocation, and binding to androgen response elements (AREs). This process regulates androgen‐responsive gene expression. In the hair follicle, this promotes progressive follicular miniaturization and shortening of the anagen phase (Lolli et al. 2017). Pharmacological treatment strategies for androgen‐related diseases primarily target androgen signaling pathways and include agents such as 5α‐reductase inhibitors (e.g., finasteride) and antiandrogenic compounds such as bicalutamide (Fernandez‐Nieto et al. 2020; Gomez‐Zubiaur et al. 2023; Kaufman et al. 1998). By competitively blocking the androgen receptor, nonsteroidal antiandrogens inhibit androgen signaling in target tissues. As a consequence of reduced androgen receptor–mediated feedback, circulating levels of luteinizing hormone and androgens may increase (Furr and Tucker 1996). This can further influence urinary ratios containing testosterone and its metabolites.

In antidoping analysis, urinary concentrations of testosterone (T) and epitestosterone (EpiT) and related metabolites, including androsterone (A), etiocholanolone (Etio), 5α‐androstane‐3α,17β‐diol (5αAdiol), and 5β‐androstane‐3α,17β‐diol (5βAdiol) excreted in the free, glucuronide and sulfate fractions, as well as their respective ratios, such as T/E, A/T, A/Etio, 5αAdiol/5βAdiol, and 5αAdiol/E, are routinely monitored as components of the steroid profile (SP) and, thereby, of the concept of the Athlete Biological Passport (ABP) (Mareck et al. 2008; WADA 2021b). As the SP exhibits both interindividual and intraindividual variability, each result is compared with the athlete's own longitudinal profile to enable more sensitive detection of atypical changes (Ayotte 2010; WADA 2021a).

Alterations in androgenic activity that influence endogenous steroid production, metabolism, or excretion may affect the SP parameters. This can lead to atypical passport findings and trigger further investigations, even when the causative compound itself is not explicitly prohibited by the World Anti‐Doping Agency (WADA). As a result, substances interfering with androgen metabolism or signaling may represent a potential confounding factor in SP interpretation (Kuuranne et al. 2014).

One substance interfering with the androgen signaling is RU 58841, also known as PSK‐3841 or HMR‐3841. This nonsteroidal antiandrogen was originally developed in the 1980s as an AGA, acne, and hirsutism therapeutic (Battmann et al. 1994). In contrast to conventional androgen pathway–targeting substances, RU 58841 acts as an androgen receptor antagonist, administered topically. Despite the lack of regulatory approval, RU 58841 remains accessible through numerous online suppliers. The increasing availability and potential use of such products raise questions regarding their systemic exposure and their potential impact on routine doping controls, particularly the SP and anabolic agent testing protocols. Preclinical studies suggested limited systemic exposure following topical administration (Pan et al. 1998). Despite these characteristics, the influence of RU 58841 on the human SP has not been investigated yet.

The present study aimed to characterize the human metabolism of RU 58841 for the first time and to assess its systemic exposure following topical application. Potential metabolites were predicted in silico, both in vitro and in vivo metabolism studies were conducted, and the intact drug as well as tentatively identified metabolites were measured using liquid chromatography–high‐resolution (HRMS) and low‐resolution (MS) (tandem) mass spectrometric approaches. In order to assess the absorption and the elimination behavior of RU 58841 and its metabolites, dried blood spot (DBS) and urine samples were collected prior to and after a single dose drug application. In addition, urine samples were analyzed by gas chromatography (GC)‐MS/MS to investigate the potential effects of RU 58841 on the urinary SP.

## 2 Experimental

### 2.1 Chemicals and Materials

Reference material of RU 58841 was obtained from Hycultec (Beutelsbach, Germany), andarine from Toronto Research Chemicals (Toronto, Canada) and testosterone‐d3 from Sigma‐Aldrich (Burlington, MA, USA). Ultrapure water was generated in‐house with a BarnsteadGenPure xCAD Plus from Thermo Scientific (Bremen, Germany). As internal standard (ISTD), a 500‐ng/mL solution of testosterone‐d_3_ and andarine in methanol was used. Acetonitrile (ACN) was obtained from VWR chemicals (Radnor, PA, USA). Methanol was purchased from J.T. Baker by Avantor (Radnor, PA, USA). Formic acid (FA) was purchased from Carl Roth GmbH + Co. KG (Karlsruhe, Germany).

For the in vitro experiment, human liver S9 (HLS9) fraction and human liver microsomes (HLM; 20 mg/mL, Thermo Fischer Scientific [Waltham, MA, USA]), 3′‐phosphoadenosine‐5′‐phosphosulfate (PAPS) tetralithium salt (Merck KGaA [Dresden, Germany]), D‐saccharic acid 1,4‐lactone monohydrate (SL; Sigma‐Aldrich; Burlington, MA, USA) and uridine diphosphate‐glucuronic acid (UDPGA; Merck KGaA [Dresden, Germany]) were used. The nicotinamide adenine dinucleotide phosphate (NADPH)‐regenerating system was obtained from PROMEGA (Madison, WI, USA). Hydrogen phosphate and magnesium chloride for the in vitro buffer were purchased from Sigma‐Aldrich (Burlington, MA, USA).

For all volume measurements, research plus pipettes from Eppendorf (Hamburg, Germany) with a maximum deviation of ±1% were used. Urine sample preparation was conducted with solid‐phase extraction (SPE) columns (CHROMABOND HLB, 60 μm, 3 mL/60 mg) from Macherey Nagel (Düren, Germany). For capillary blood sampling, Solofix Safety Sterile, single‐use safety lancet from B. Braun (Melsungen; Germany) and DBS cards (QIAcard FTA DMPK‐C) from QIAGEN (Hilden, Germany) were used. The administration study was performed with 5% RU 58841 purchased via the internet, which was tested for purity before use.

### 2.2 Instruments

For metabolite identification, LC‐HRMS analysis was performed on a Vanquish UHPLC system coupled to an Orbitrap Exploris 480 mass spectrometer (Thermo Fisher Scientific; Bremen, Germany). For chromatographic separation, a Poroshell 120 EC‐C18 column (3.0 × 50 mm, 2.7 μm; Agilent Technologies; Santa Clara, CA; USA) in combination with an EC 4/3 Nucleoshell RP‐18 Plus pre‐column (Macherey Nagel; Düren, Germany) was chosen. For LC separation, eluent A consisted of 0.1% (v/v) formic acid (FA) in H_2_O, while eluent B consisted of 0.1% (v/v) FA in ACN. The gradient started at 10% B and was maintained isocratic for 1 min, followed by a linear increase to 60% B over the next 5 min. The proportion of eluent B was then increased to 100% by 7 min and held isocratic for 1 min as a column washing step. Subsequently, the column was re‐equilibrated to the initial conditions for 3 min. The flow rate was maintained at 0.4 mL/min, and an injection volume of 5 μL was used. Separate acquisition methods with identical LC conditions were applied for positive and negative electrospray ionization modes with an ion source temperature of 420°C. The ion transfer tube was maintained at 320°C. Targeted MS/MS experiments were performed using a resolving power of 60,000 FWHM and an isolation window of *m/z* 1.

LC–MS/MS analyses were performed on an ACQUITY I‐Class ultra‐performance liquid chromatograph (UPLC) coupled via ZSpray electrospray ionization (ESI) source in both positive and negative ionization modes to a Xevo Triple Quadrupole‐XS mass spectrometer (TQ‐XS‐MS; Waters Eschborn, Germany). Samples prepared for analysis were injected onto a Nucleodur C‐18 pyramid analytical column (50 × 3.0 mm, 1.5 μm particle size; Agilent, Waldbronn, Germany). The injection volume was set at 5 μL and the gradient was applied with a flowrate of 0.4 mL/min using 0.1% FA as eluent A and 0.1% FA in ACN as eluent B. The gradient started at 10% B and was isocratic for 0.5 min, increasing to 60% B over 6.5 min, followed by an increase to 95% B over 1 min, and was then flushed at 95% B for 3 min followed by re‐equilibration at 10% B for 3 min. The analyses were conducted using time‐based multiple‐reaction monitoring (MRM). Respective ion transitions, collision energies, and retention times are shown in Table 1. The data were processed using TargetLynx (Waters; Eschborn, Germany).

**TABLE 1:** LC–MS/MS acquisition parameters for RU 58841 and its metabolites (M1–M6), including retention times (RT), cone voltages, precursor‐to‐product ion transitions, and collision energies (ce).

Compound	Ion species	RT (min)	Cone voltage (V)	*m*/*z* transitions (exp.)	ce (eV)
RU 58841	[M + H]^+^	5.97	20	370 > 70/310	50/20
M1	[M + H]^+^	5.98	20	386 > 71/368	40/20
M2/cyanonilutamide	[M − H]^−^	6.10	20	296 > 253/185	40/40
M3	[M − H]^−^	6.09	20	382 > 253/296	50/30
M4	[M − H]^−^	5.34	20	544 > 253/296	50/30
M5	[M − H]^−^	5.48	20	560 > 253/296	50/30
M6	[M − H]^−^	5.48	20	558 > 253/296	50/30

### 2.3 Urine Sample Preparation

For sample preparation, 1 mL of urine was mixed with 10 μL of the ISTD solution. For SPE, cartridges were first pre‐conditioned with 3 mL of methanol and 3 mL of water, and then loaded with the urine sample. After washing the cartridges with 2 mL of water, elution was performed with 1 mL of methanol. The eluate was dried under a nitrogen stream at 50°C and reconstituted with 100 μL of H_2_O/ACN (90:10; v/v).

For the determination of the SP parameters, the accredited routine doping control method of the Institute of Biochemistry at the German Sport University Cologne was used (Mareck et al. 2008; Thevis et al. 2011).

### 2.4 Dried Blood Spot Sample Preparation

Capillary blood samples were collected from the fingertip using a 20 μL capillary tube. The tube was emptied onto a DBS filter card and dried for at least 3 h at room temperature. Subsequently, the DBS cards were stored individually in Ziploc plastic bags with a desiccant silica gel at −20°C until analysis.

The entire DBS was cut out and extracted with 1 mL of methanol. Ten microliters of the ISTD solution was added to the tubes. After sonication for 30 min, the supernatant was transferred into a fresh tube, dried under a nitrogen stream at 50°C, and reconstituted with 100 μL of H_2_O/ACN (90:10; v/v). The samples were finally centrifuged at 17,000 × *g* for 10 min, and the supernatant was transferred to glass vials before analysis.

### 2.5 Validation

Due to the lack of reference material of the metabolites, the full method validation for urine and DBS was conducted with RU 58841 only; however, selectivity was tested for all substances. Both methods were validated according to the requirements of current WADA documents, including the parameters selectivity, limit of detection (LOD), limit of identification (LOI), recovery, linearity, stability, precision, matrix effects, and carry‐over (WADA 2021c).

For urine samples, the previously described SPE method was validated by spiking urine with the analyte at defined concentrations. For the DBS method, DBS from spiked whole blood in ethylenediaminetetraacetic acid (EDTA)–coated tubes was used. Selectivity of the methods was confirmed by measuring 10 samples from healthy volunteers (five males, five females), probing for interfering signals (aimed detection rate 0/10). The LOD was estimated via the detection rate of six samples from different volunteers fortified at concentrations of 0.2, 0.5, 1, 2, 5, 6, and 8 ng/mL in urine samples, and at concentrations of 0.5, 0.8, 1, 3, 5, and 10 ng/mL in DBS samples. The criteria for detection were retention time (RT) compared to a reference standard and a signal‐to‐noise ratio (S/N) greater than 3 for the extracted ion chromatogram of the quantifier ion. The LOD was estimated as the concentration at 95% analyte detection rate. The LOI was evaluated based on RT and relative abundances of two product ions compared to a reference standard. A detection rate of 95% was applied as acceptance criterion. For the evaluation of the interday imprecision, samples were prepared at three concentration levels. In urine, the concentration levels 10, 50, and 100 ng/mL, and in DBS, the concentration levels 5, 20, and 100 ng/mL were used. Each concentration level was prepared and analyzed in three independent batches on different days. The samples were processed according to the same sample preparation procedure and analyzed under identical instrumental conditions. Interday imprecision was assessed by calculating the relative standard deviation (%RSD) of the measured concentrations obtained from all batches at each concentration level. Intraday imprecision describes the %RSD in one batch.

To investigate the recovery, six samples were fortified before extraction and six samples after the extraction at a concentration of 10 ng/mL in urine and 5 ng/mL in DBS. Recovery was computed as the loss of target analyte during the sample preparation procedure in %.

The extract stability in the autosampler was tested by measuring a batch of samples and remeasuring the same batch after storage in the autosampler at 10°C for 72 h. Carry‐over in the LC–MS/MS system was tested by measuring samples of high target analyte concentration (200 ng/mL) and two subsequent blank samples, assessing peak areas (if detected) in the chromatograms of the blank urine specimens. The linearity of the method was examined by measuring 7 samples of increasing concentration (0–200 ng/mL) and plotting the ratio of the measured analyte area to the ISTD area against the concentration. Matrix effects were assessed by comparing the analyte responses obtained in the presence of biological matrix components with those from corresponding samples prepared in water at a concentration level of 10 ng/mL in urine and 20 ng/mL in DBS. For urine analysis, the analyte was spiked into water and the obtained responses were compared with those from four urine samples spiked with the analyte. Water and urine samples were extracted according to the described procedure. For DBS analysis, four samples were prepared by spiking the analyte into water and pipetting the solution on DBS cards. The responses obtained from these samples were compared with those from six blood samples spiked with the analyte and processed under the same conditions.

### 2.6 In Silico Metabolite Prediction

Potential metabolites of RU 58841 were predicted using their SMILES string obtained in PubChem with an open‐access software tool BioTransformer 3.0. The software predicts biotransformation products by applying rule‐based metabolic transformation pathways.

### 2.7 Sample Preparation In Vitro Metabolism Experiment

Standard protocols were followed for in vitro metabolism experiments as reported elsewhere (Kuuranne et al. 2008), with slight modifications. Briefly, substrate sample, substrate‐blank and enzyme‐blank were prepared in an incubation buffer, which includes 50 mM phosphate buffer (pH 7.4) containing 5 mM of magnesium chloride. For phase I metabolism, 10 μL of RU 58841 (100 μM), 10 μL of NADPH‐regenerating system (50 mM), 5 μL of HLS9 fraction (20 mg/mL), and 5 μL of HLM (20 mg/mL) were added to 20 μL of phosphate buffer into 1.5 mL low‐bind microcentrifuge tubes. The mixtures were incubated for 20 h at 37°C and 500 rpm.

For phase II metabolism, 5 μL of HLS9 fraction, 5 μL of HLM fraction, as well as 10 μL of UDGPA (50 mM), 10 μL of SL (50 mM) and 10 μL of PAPS (20 μM) were added into an incubation solution after completion of phase I reactions. The mixture was heated for an additional 24 h at 37°C and agitation at 500 rpm.

After incubation, 10 μL of the ISTD solution were added and the reaction was terminated by addition of 150 μL of ice‐cold ACN, followed by vortex and incubation on ice for 10 min. After centrifugation at 17,000 × *g* for 5 min, the supernatant was transferred to a fresh test tube and the solvent evaporated in a centrifuge under reduced pressure. The samples were then reconstituted in 10% ACN in H_2_O before subjecting the sample to analysis. The prepared substrate‐blank contained all components except RU 58841. It accounts for any background signal caused by the enzyme itself, such as intrinsic absorbance or interfering compounds. The enzyme‐blank contained RU 58841 but lacked enzymatic source (HLM and S9 fraction). It corrects for any signal originating from the substrate, such as spontaneous degradation or background absorbance. Subtraction of these controls ensures that the measured response specifically reflected enzymatic conversion of the substrate. The in vitro experiment was performed in triplicate.

### 2.8 Product Purity Test

The concentration of the 5% RU 58841 hair liquid serum, purchased via the internet, was estimated by standard addition analysis. Therefore, the product was diluted by a factor 500,000 in H_2_O:ACN (1:1; v:v). After adding 100, 150, 200, or 300 ng of a RU 58841 standard solution to aliquots of the diluted product, and further 10 μL of the ISTD solution, the aliquots were topped‐up to a total volume of 1 mL with H_2_O/ACN (90:10; v/v) for LC–MS/MS analysis. The experiment was performed in triplicate.

### 2.9 Administration Study

The administration study was approved by the local ethics committee of the German Sport University Cologne (#266/2025), and written informed consent was obtained from all participants. The excretion study was conducted with six healthy male volunteers. Prior to administration, blank urine (three specimens) and one DBS sample were collected to confirm the absence of target analytes prior to administration. In addition, the blank urine samples were used for the determination of baseline SP parameters. The study protocol involved a single administration of 1 mL of a RU 58841‐containing hair serum formulation, equaling an application of 30 mg of the androgen receptor antagonist.

Blood and urine samples were collected for 7–10 days. Within the first 24 h, each urine sample was collected. Subsequently, three urine samples per day were collected between 24–48 h post‐administration, and two samples per day after 48 h. DBS samples were obtained at 3, 6, 9, 12, and 24 h post‐administration. After 24 h, three DBS samples were collected per day; after 48 h, two samples per day were collected; and after 72 h, sampling was restricted to morning collection only. Two DBS spots were collected at each sampling time point.

## 3 Results and Discussion

### 3.1 Validation

The analytical methods for the determination of RU 58841 in urine and DBS samples were comprehensively validated with respect to specificity, LOD, LOI, precision, stability, recovery, matrix effects, linearity, and carry‐over (Table 2). No interfering signals were observed in 10 blank urine and DBS samples, demonstrating adequate specificity. The LODs were determined with 7 ng/mL in urine and 0.8 ng/mL in DBS. Urine extract stability experiments (storage for 72 h in the autosampler at 10°C) revealed a signal recovery of 56% at 10 ng/mL, indicating limited analyte stability under the investigated conditions. In contrast, DBS sample extracts returned signal abundance at 85% under same conditions. Both methods were found to be linear from the LOD to 200 ng/mL, and no carry‐over was detected in this range. The obtained values for the urine and DBS methods were within acceptable limits and were considered suitable for the intended application.

**TABLE 2:** Validation parameters of the analytical method for RU 58841 in DBS and urine.

Parameter	*n*	c (ng/mL)	DBS	c (ng/mL)	Urine
Specificity	10	—	✓	—	✓
Carry‐over	1	200	0%	200	0%
Linearity	8	0.8–200	*R* ^2^ = 0.9954	8–200	*R* ^2^ = 0.9786
LOD	6	—	0.8 ng/mL	0.2–10	7 ng/mL
LOI	6	—	10 ng/mL	0.2–10	9 ng/mL
Intraday‐imprecision	18	5	11%	10	13%
	18	20	10%	50	13%
	18	100	10%	100	14%
Interday‐imprecision	6	5	9%–11%	10	11%–12%
	6	20	4%–11%	50	9%–18%
	6	100	5%–13%	100	12%–17%
Stability after 72 h	6	20	85%	10	56%
Recovery	5	5	92%	10	97%
	6	100	99%		
Matrix effects	6	20	88%	10	51%

### 3.2 In Vitro and In Silico Metabolism Experiments

**FIGURE 1:**
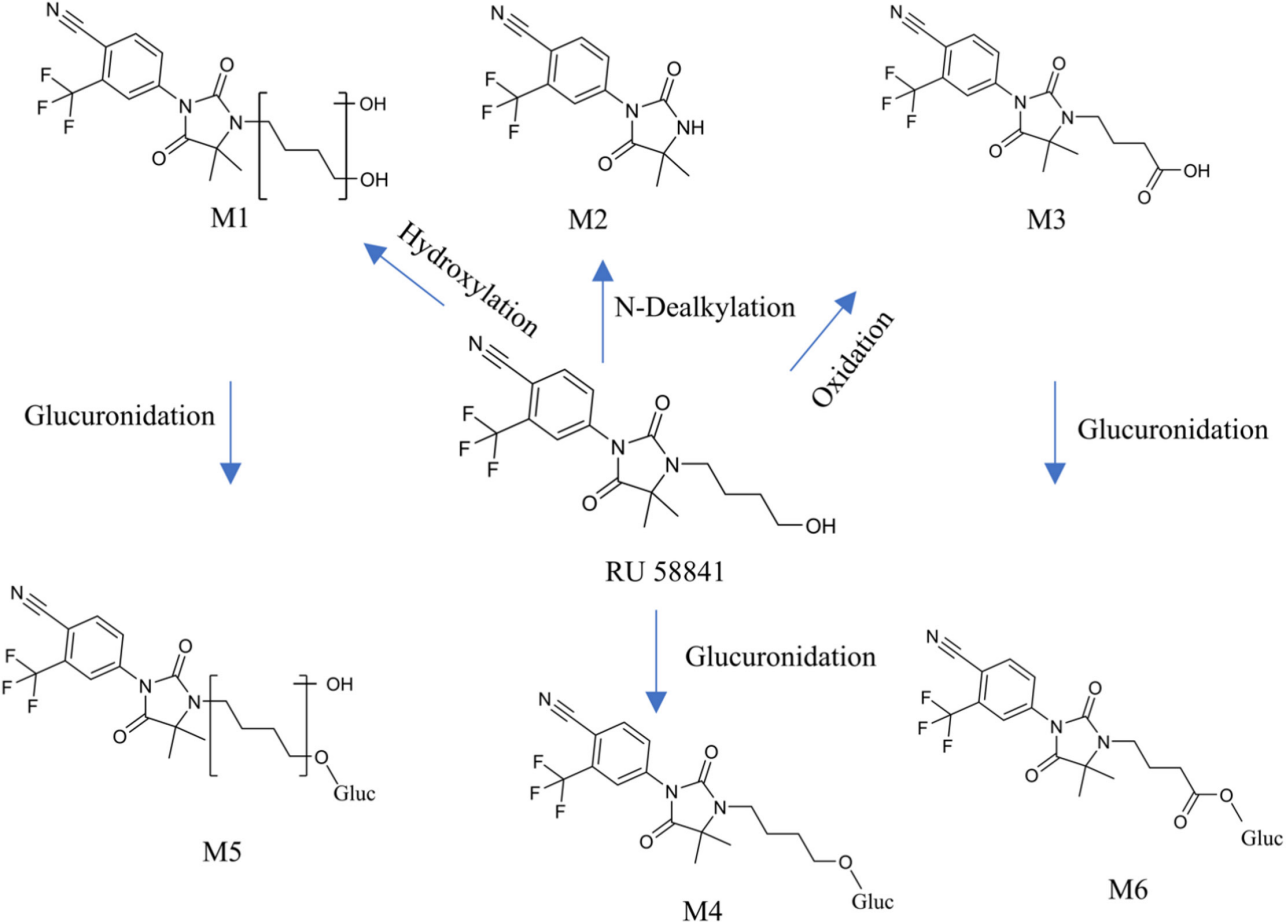
Overview of the proposed in vitro metabolic pathways of RU 58841.

In this study, an in vitro metabolism investigation was performed to obtain an initial overview of potential metabolites of RU 58841. Following incubation with pooled HLM and HLS9 fraction, six metabolites were identified using HRMS (Figure 1). The product ions of each metabolite are summarized in Table 3, while the corresponding MS/MS spectra and proposed structures are provided in the Supplementary Information. Metabolites M2 and M3 were reported previously in an animal study by Battmann et al. (1994).

**TABLE 3:** Elemental compositions of protonated or deprotonated molecules of RU 58841 and M1–M6 with resulting diagnostic product ions using high resolution MS/MS experiments.

	Metabolic reaction	*m*/*z*	Elemental composition	Error (ppm)	RT (min)	Product ions (*m*/*z*)	Elemental composition	Error (ppm)	ce (eV)
RU 58841	—	370.1373	C_17_H_19_F_3_N_3_O_3_^+^	2.4	5.97	352.1287	C_17_H_17_F_3_N_3_O_2_^+^	2.3	30
						310.0815	C_14_H_11_F_3_N_3_O_2_^+^	2.3	
						70.0651	C_4_H_8_N^+^	2.2	
M1	Hydroxylation	386.1322	C_17_H_19_F_3_N_3_O_4_^+^	2.6	5.98	368.1217	C_17_H_17_F_3_N_3_O_3_^+^	3.4	30
						350.1111	C_17_H_15_F_3_N_3_O_2_^+^	3.9	
						338.1111	C_16_H_15_F_3_N_3_O_2_^+^	3.8	
						310.0798	C_14_H_11_F_3_N_3_O_2_^+^	3.8	
						298.0798	C_13_H_11_F_3_N_3_O_2_^+^	4	
						110.0964	C_7_H_12_N^+^	2.5	
						98.0964	C_6_H_12_F_3_N^+^	3.2	
						71.0491	C_4_H_7_O^+^	2.6	
M2	N‐dealkylation	296.0652	C_13_H_9_F_3_N_3_O_2_^−^	0.7	6.1	253.0594	C_12_H_8_F_3_N_2_O^−^	1.3	40
						185.0332	C_8_H_4_F_3_N_2_^−^	1.3	
M3	Oxidation	382.102	C_17_H_15_F_3_N_3_O_4_^−^	1.8	5.98	296.0652	C_13_H_9_F_3_N_3_O_2_^−^	1.9	40
						253.0594	C_12_H_8_F_3_N_2_O^−^	1.9	
						185.0332	C_8_H_4_F_3_N_2_^−^	1	
M4	Glucuronidation	544.1548	C_23_H_25_F_3_N_3_O_9_^−^	0.3	5.34	484.1337	C_21_H_21_F_3_N_3_O_7_^−^	0.5	35
						426.1282	C_19_H_19_F_3_N_3_O_5_^−^	2.4	
						368.1227	C_17_H_17_F_3_N_3_O_3_^−^	3.2	
						296.0652	C_13_H_9_F_3_N_3_O_2_^−^	1.4	
						253.0594	C_12_H_8_F_3_N_2_O^−^	0.9	
						185.0332	C_8_H_4_F_3_N_2_^−^	1.2	
						113.0244	C_5_H_5_O_3_^−^	1.4	
						85.0295	C_4_H_5_O_2_^−^	0.8	
						71.0139	C_3_H_3_O_2_^−^	0.2	
M5	Hydroxylation + glucuronidation	560.1498	C_23_H_25_F_3_N_3_O_10_^−^	0.6	5.48	442.1227	C_19_H_19_F_3_N_3_O_6_^−^	1	40
						384.1177	C_17_H_17_F_3_N_3_O_4_^−^	2.5	
						296.0652	C_13_H_9_F_3_N_3_O_2_^−^	1	
						253.0594	C_12_H_8_F_3_N_2_O^−^	0.8	
						185.0332	C_8_H_4_F_3_N_2_^−^	1.1	
						113.0244	C_5_H_5_O_3_^−^	0.4	
						85.0295	C_4_H_5_O_2_^−^	1.4	
						71.0139	C_3_H_3_O_2_^−^	0.2	
M6	Oxidation + glucuronidation	558.1341	C_23_H_23_F_3_N_3_O_10_^−^	1.2	5.48	382.102	C_17_H_15_F_3_N_3_O_4_^−^	2.1	20
						296.0652	C_13_H_9_F_3_N_3_O_2_^−^	1.3	
						193.0354	C_6_H_9_O_7_^−^	1.5	
						175.0248	C_6_H_7_O_6_^−^	1.7	
						157.0142	C_6_H_5_O_5_^−^	0.7	
						131.035	C_5_H_7_O_4_^−^	0.8	
						113.0244	C_5_H_5_O_3_^−^	0.4	

Starting from the parent compound RU 58841, three phase I metabolic pathways were identified, predominantly mediated by cytochrome P450 (CYP) enzymes. Metabolite M1 is formed by hydroxylation of the parent compound. Cleavage of the hydroxybutyl side chain yields metabolite M2 (cyanonilutamide), whereas oxidation of the terminal hydroxyl group to a carboxylic acid resulted in the formation of metabolite M3. The latter reaction is typically catalyzed by alcohol and aldehyde dehydrogenases.

In addition to phase I biotransformations, RU 58841 undergoes phase II metabolism via glucuronidation. Glucuronidation of the parent compound resulted in the formation of M4, while conjugation of metabolites M1 and M3 produced M5 and the acyl glucuronide M6, respectively. Acyl glucuronides constitute a distinct class of glucuronides characterized by their high chemical reactivity and are thought contribute to drug toxicity, but direct evidence for their toxicity in vivo is scarce (Ritter 2000; Shipkova et al. 2003). However, the reactivity of acyl glucuronides varies considerably between compounds. More stable acyl glucuronides generally exhibit longer degradation half‐lives than more reactive analogues (Shipkova and Wieland 2005). However, for future testing, it is advisable to assess the stability of M6 in urine samples during transport and storage.

An in silico metabolism study was performed using BioTransformer 3.0, which successfully predicted the formation of metabolites M1–M6. In addition, the oxidation of the terminal hydroxyl group to the corresponding aldehyde intermediate, followed by further oxidation to the corresponding carboxylic acid was postulated. However, only the carboxylic acid (M3) was detected in in vitro experiments, whereas the aldehyde intermediate was not observed. Furthermore, metabolites resulting from combinations of hydroxylation and oxidation, as well as the formation of 4‐hydroxybutanal and sulfation of hydroxyl groups were suggested using the in silico prediction tool. However, none of these metabolites were eventually detected in samples analyzed after in vitro incubation of RU 58841. Overall, the in silico metabolism experiment provided a comprehensive overview of potential metabolites and enabled the generation of a preliminary target list for metabolite identification in subsequent in vitro and in vivo studies.

### 3.3 Purity Test

For the administration study, a hair serum containing alcohol, water, propylene glycol and RU 58841, obtained from an internet‐based supplier, was used. The RU 58841 concentration listed on the product was 50 mg/mL. The determined concentration via standard addition was 30 mg/mL, and no other drugs were detected.

### 3.4 Administration Study

The aim of the study was to evaluate the systemic exposure of RU 58841 after topical administration of 30 mg of RU 58841 and to evaluate the metabolic fate of RU 58841 in humans. Therefore, the collected DBS and urine samples were analyzed for the intact parent compound and metabolites M1–M6. In addition, LC‐HRMS analyses were performed using a target analyte list considering the in silico metabolism‐derived biotransformation products After evaluating the elimination profile of RU 58841 and its metabolites, the influence of the administration on the SP was investigated.

#### 3.4.1 Administration Study: Metabolite Confirmation In Vivo

**FIGURE 2:**
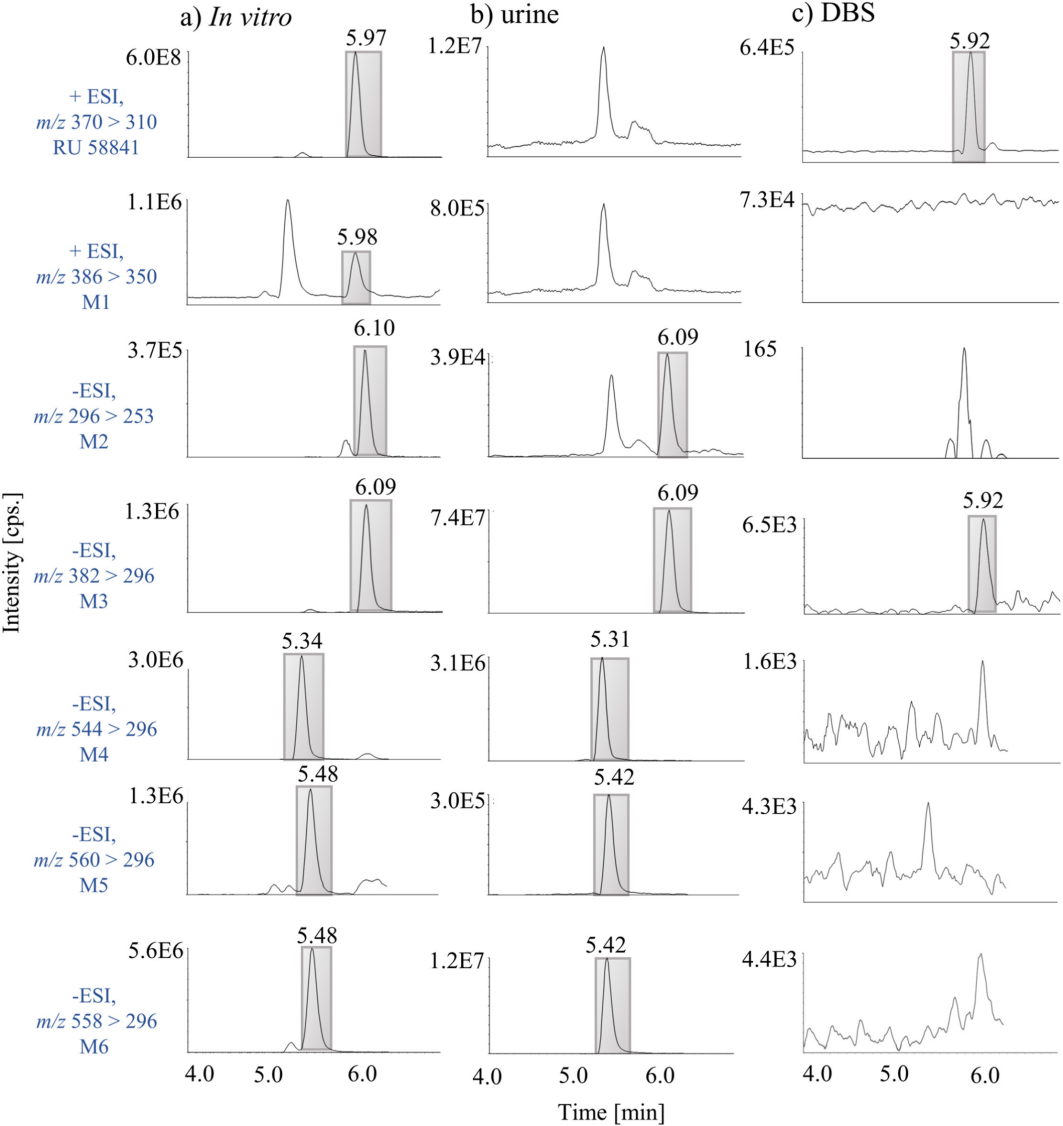
Extracted ion chromatograms of RU 58841 and its metabolite M1–M6 in (a) an in vitro incubation experiment, (b) in a urine sample (3 h post‐application), and (c) in a DBS sample (12 h post‐application).

The collection of urine and DBS samples prior to the administration confirmed that the metabolites and the intact parent compound were not detectable before administration. After administration, five metabolites (M2–M6) were detectable in urine samples, whereas the parent compound was not observed. In contrast, DBS samples allowed for the detection of the intact parent compound as well as M3 (Figure 2).

RU 58841 exhibited a RT of 5.97 min in the quality control samples. Accordingly, the intact parent compound was detected in the in vitro incubation and DBS samples. In urine, only a peak at RT 5.32 min was observed, which was also present in the in vitro samples. As this RT corresponds to that of the glucuronidated metabolite, the signal was attributed to an in‐source fragmentation‐derived product of M4. Likewise, for M1, a signal at RT 5.36 min was detected in the urine sample, which likely corresponds to an in‐source fragment of M5. In the samples of the in vitro incubation experiment, a peak at RT 5.98 min was observed corresponding to M1 itself. M2 was detected in the in vitro and urine samples, exhibiting a RT of 6.10 min. M3 (RT = 6.09 min) was the only metabolite consistently detected in the in vitro, urine, and DBS samples. Phase II metabolites were not detectable in DBS samples. The glucuronidated parent compound eluted at RT 5.34 min in the in vitro and urine samples. Metabolites M5 and M6 were also detected in vitro and in urine, eluting at RT 5.48 min respectively. As M2 was not detectable in vivo, this compound was not further investigated. To determine the detection windows, excretion profiles of each metabolite were recorded.

#### 3.4.2 Administration Study: Elimination Profile Urine

**FIGURE 3:**
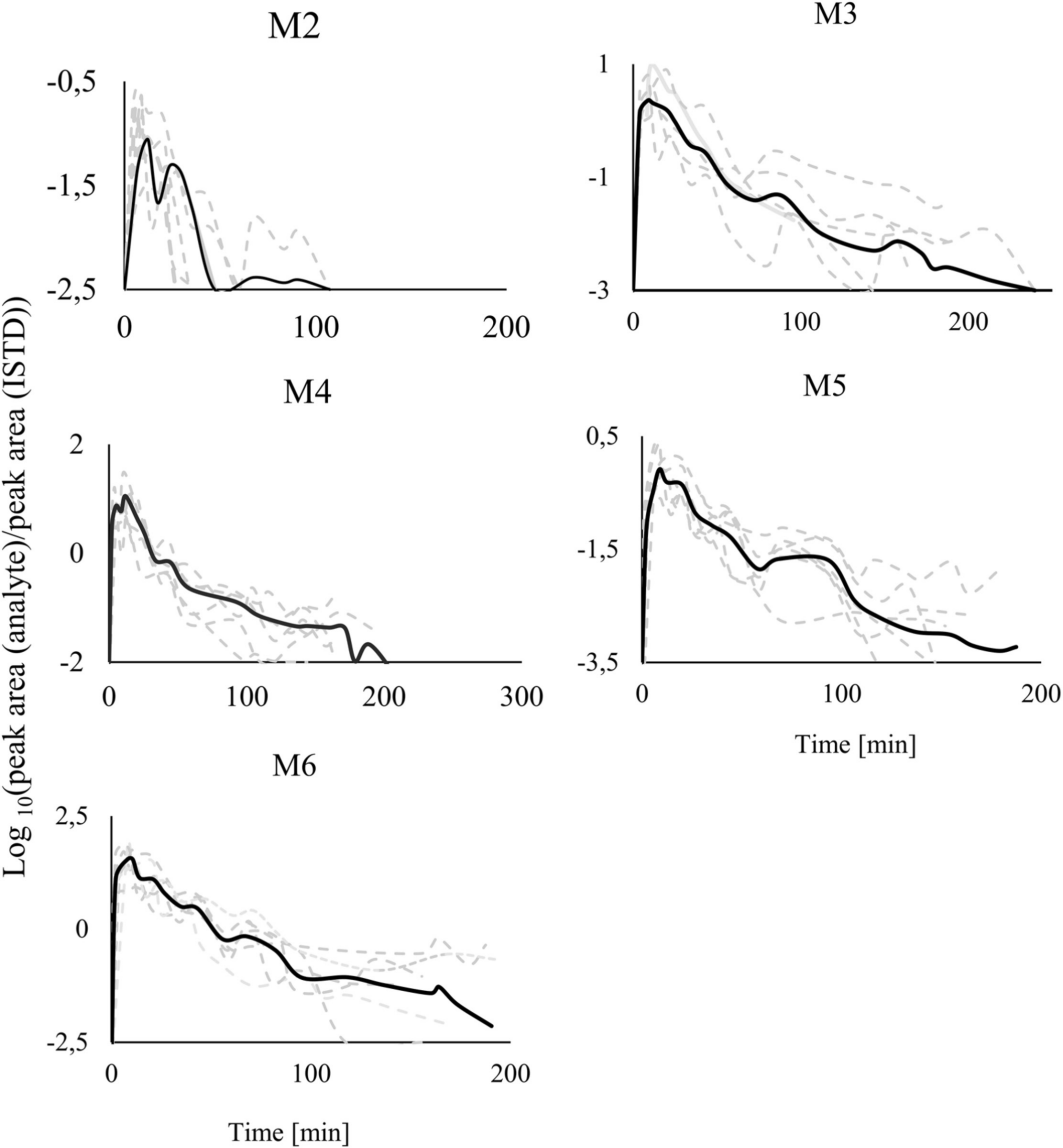
Urinary excretion profiles of M2–M6 after topical single dose administration of 30 mg RU 58841; black solid lines represent the mean analyte levels across all participants. Individual elimination profiles are depicted as gray dashed lines; *x*‐axis intercept = substance was not detected.

After the administration of 30 mg of RU 58841 on the hair scalp of six male participants, M2–M6 were detectable in urine samples. Their excretion profiles are shown in Figure 3. Due to the lack of reference material, the metabolites were not quantified and shown as the logarithm of the ratio between the analyte and the ISTD. Phase I metabolites M2 and M3 exhibited a shorter detection window compared to the phase II metabolites. M2 was detectable for up to 108 h post‐administration. In most participants, however, the detection window ranged between approximately 30 and 60 h. M3 exhibited a substantially longer detection window, enabling an identification up to 213 h in one participant. For M4, one participant tested negative after 140 h, whereas urine samples of all other participants returned findings for M4 until the final sample was collected (165–213 h). Similarly, M5 was no longer detectable after 156 h in one participant, while it remained visible in all other participants through the end of the sampling period. Long detection windows are generally desirable in doping controls, as they increase the probability of uncovering the use of a prohibited substance. In the present study, the phase II metabolites M4–M6 exhibited the longest detection windows and therefore represent the most suitable targets for long‐term monitoring of RU 58841 intake. Despite the generally recognized chemically labile nature of acyl glucuronides, the metabolite remained detectable in urine for approximately 200 h after administration. This prolonged detection window suggests that the acyl glucuronide is sufficiently stable under the investigated conditions, although dedicated stability studies would be required to confirm its intrinsic chemical stability. Metabolites with shorter detection windows, such as M2 and M3, may also provide valuable information, as their presence may indicate a more recent administration. Especially in the context of presumed contamination scenarios, such information can assist in distinguishing between a recent low‐dose exposure and an earlier administration of a therapeutically relevant amount.

#### 3.4.3 Administration Study: Elimination Profile in DBS

**FIGURE 4:**
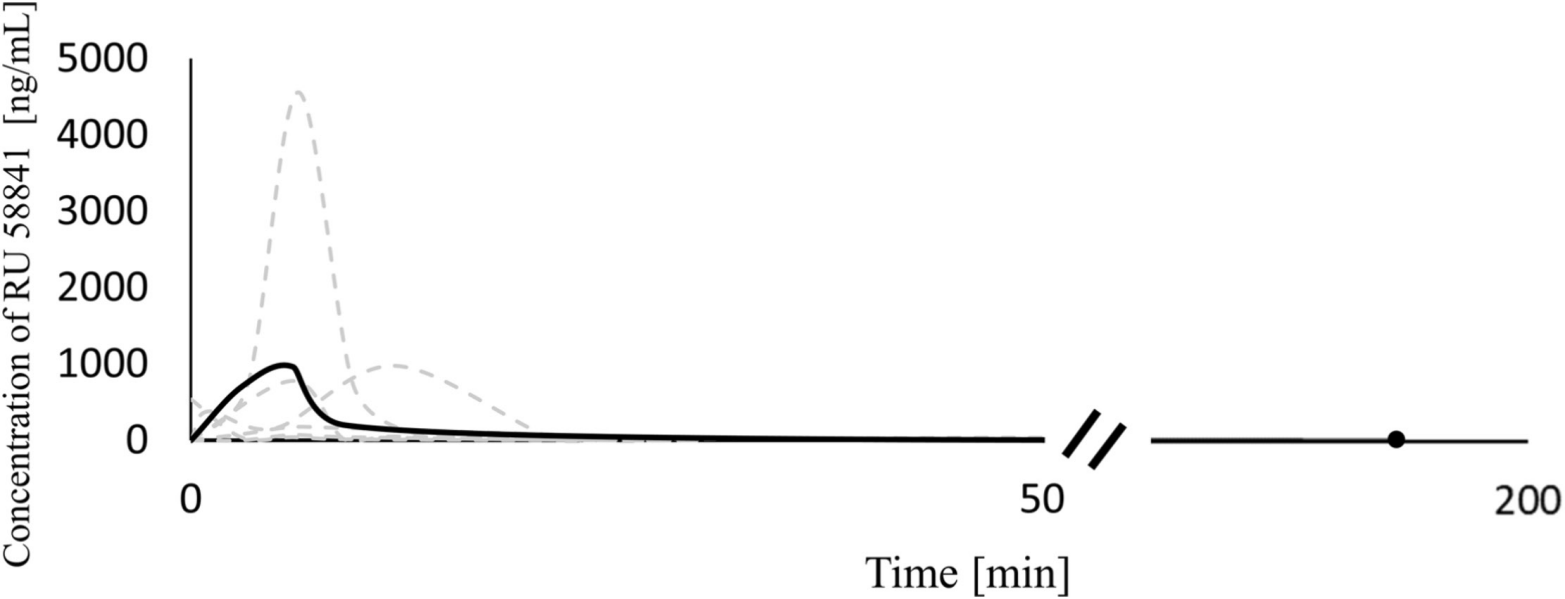
Elimination profile of RU 58841 in DBS following a topical administration of 30 mg of RU 58841. The black solid line represents the mean concentration across all participants. Individual elimination profile depicted as a gray dashed line. The dot symbolizes one sample that still allowed for identifying RU 58841.

The intact parent compound RU 58841 was not detected in urine samples after administration. However, it was detectable in DBS samples from all six participants up to 168 h after administration (Figure 4). The concentrations of RU 58841 increased rapidly and reached maximum levels within the first 12 h post‐dose. The highest concentration was observed with ca. 5 μg/mL. As this concentration exceeded the validated calibration range, a second calibration curve was prepared from 1 to 10 μg/mL and tested for linearity (*R* ^2^ = 0.9563). This calibration curve was applied directly to the measured peak areas without re‐extraction of the DBS. Concentrations decreased to near‐baseline levels by 36 h. After 120 h, only one participant returned findings of low concentrations (<LOI) of RU 58841 in DBS. This indicates that RU 58841 is systemically available after topical administration and therefore has the potential of exhibiting systemic effects. Additionally, M3 was detectable within the first 12 h in DBS samples (data not shown). These shorter detection windows, compared with those of the urinary metabolites, may provide additional information regarding the timing and dose of an administration.

With six study volunteers, this exploratory study cannot be considered as fully capturing interindividual variability in the metabolism of RU 58841. However, as the primary objective of this study was to identify human metabolites and to characterize their elimination profiles, essential information was obtained that allows for including markers for RU 58841 use in an antidoping context, if required.

#### 3.4.4 Administration Study: Influence on the SP

**FIGURE 5:**
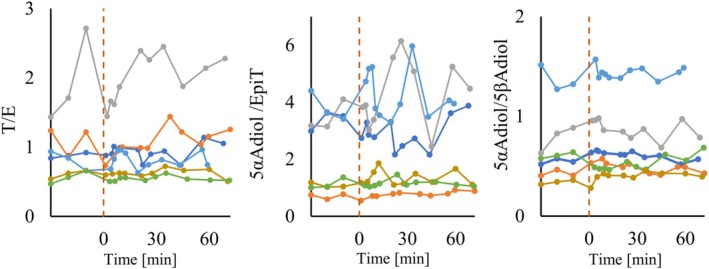
Ratios of urinary concentrations of T/E, Adiol/EpiT, and 5α Adiol/5β Adiol in all six subjects.

In the present study, the effects of a single dose administration of 30 mg of RU 58841 on the human SP parameters were investigated. Three urine samples collected prior to administration were analyzed to establish individual baseline values for each participant. All ratios relevant for the SP were evaluated throughout the study. As representative examples, the urinary concentration ratios of T/E, 5αAdiol/EpiT, and 5αAdiol/5βAdiol are shown in Figure 5, as they are known to be the most sensitive for the testosterone intake (Mullen et al. 2018). Deviations in these ratios beyond the individual thresholds may trigger further confirmatory investigations during doping controls and may ultimately contribute to the reporting of an Adverse Analytical Finding.

As only three blank values were collected, they may not show the expected variance needed for a representative SP. Nevertheless, no alterations in any of the SP markers were observed in any of the six participants following topical RU 58841 administration, indicating that a single dose does not affect the urinary SP parameters routinely evaluated in doping control. RU 58841 is generally intended for long‐term use in clinical settings. Repeated daily use may result in cumulative or steady‐state endocrine adaptations through physiological feedback mechanisms. Therefore, future longitudinal studies are necessary to determine the endocrine effects during chronic daily use.

To our knowledge, this study provides the first evidence regarding the potential effects of RU 58841 on the SP. Although the sample size was limited to six participants and is not representative of the broader athletic population, the findings suggest that RU 58841 does not exert a detectable effect on SP under the conditions investigated. Nevertheless, these findings should be regarded as preliminary and require confirmation in larger, more diverse groups. Overall, the present results provide an initial basis for future investigations into the endocrine effects of long‐term RU 58841 use.

## 4 Conclusion

RU 58841 is a nonsteroidal antiandrogen marketed online as a treatment for AGA. However, data on its systemic exposure, metabolism, and elimination in humans have not previously been reported. Owing to its antiandrogenic mechanism of action, systemic exposure to RU 58841 could potentially influence endogenous steroid metabolism, and consequently, SP markers used in antidoping testing.

Therefore, this study investigated the in silico metabolism of RU 58841 using BioTransformer 3.0 and the in vitro metabolism using HLS9 and HLM fractions. BioTransformer 3.0 predicted phase I metabolic reactions, including hydroxylation, N‐dealkylation, and oxidation, as well as phase II reactions, such as glucuronidation and sulfation of RU 58841. In addition, metabolites resulting from sequential or combined biotransformation reactions involving both phase I and phase II metabolism were predicted. Six metabolites of RU 58841 were characterized in vitro, formed by hydroxylation (M1), N‐dealkylation (M2), oxidation (M3), and glucuronidation of the parent compound (M4), as well as glucuronic acid conjugation of the hydroxylated (M5) and the oxidized (M6) phase I metabolites. These metabolites served as analytical targets in a controlled administration study in which six healthy male volunteers received a single topical dose of 30 mg of RU 58841 applied to the scalp to evaluate systemic exposure and urinary elimination.

The metabolites M2–M6 were detected in urine, allowing characterization of their urinary elimination profiles. Phase II metabolites (M4–M6) showed the longest detection windows (up to 213 h), making them the most suitable biomarkers for long‐term detection. In contrast, the phase I metabolites M2 and M3 were detectable for a considerably shorter period and may therefore indicate more recent administration. Despite the known lability of acyl glucuronides, the corresponding metabolite remained detectable for approximately 200 h, suggesting sufficient stability during urinary excretion and sample preparation. Additionally, the results demonstrate that topical administration of RU 58841 leads to systemic exposure, as RU 58841 was detected in DBS samples in considerable amounts. Nevertheless, no influence on the SP parameters was observed in any of the six participants. Therefore, these findings do not support the hypothesis that RU 58841 acts as a confounding factor of the urinary SP in antidoping analysis.

## Author Contributions

**Aylin Okutan:** methodology, validation, investigation, formal analysis, data curation, visualization, writing – original draft. **Oliver Krug:** methodology, writing – review and editing. **Gregor Fußhöller:** data curation, writing – review and editing. **Thomas Piper:** formal analysis, writing – review and editing. **Mario Thevis:** supervision, project administration, methodology, conceptualization, writing – review and editing.

## Ethics Statement

Ethical approval was granted by the ethics committee of the German Sport University Cologne (Nr. #266/2025). All volunteers gave their informed consent for participation and publication in anonymized form.

## Conflicts of Interest

The authors declare no conflicts of interest.

## Supporting information


**Figure S1:** Product ion mass spectrum generated from the precursor ion at m/z 370.1373 of RU 58841 after positive electrospray ionization and collision‐induced dissociation at 30 eV and proposed dissociation routes.
**Figure S2:** Product ion mass spectrum generated from the precursor ion at m/z 386.1322 of M1 after positive electrospray ionization and collision‐induced dissociation at 30 eV and proposed dissociation routes, which were supported by pseudo MS3 experiments.
**Figure S3:** Product ion mass spectrum generated from the precursor ion at m/z 296.0652 of M2 after negative electrospray ionization and collision‐induced dissociation at 30 eV and proposed dissociation routes.
**Figure S4:** Product ion mass spectrum generated from the precursor ion at m/z 382.1020 of M3 after negative electrospray ionization and collision‐induced dissociation at 30 eV and proposed dissociation routes.
**Figure S5:** Product ion mass spectrum generated from the precursor ion at m/z 544.1548 of M4 after negative electrospray ionization and collision‐induced dissociation at 30 eV and proposed dissociation routes.
**Figure S6:** Product ion mass spectrum generated from the precursor ion at m/z 558.1341 of M6 after negative electrospray ionization and collision‐induced dissociation at 30 eV and proposed dissociation routes, which were supported by pseudo MS3 experiments.
**Figure S7:** Product ion mass spectrum generated from the precursor ion at m/z 560.1498 of M5 after negative electrospray ionization and collision‐induced dissociation at 30 eV and proposed dissociation routes.

## Data Availability

All data included in this study are available from corresponding authors upon reasonable request.
