# Supplementary material for: Investigation Into the Metabolism and Elimination Behavior of RU 58841, and Its Influence on the Steroid Profile in Humans
Source: Biomed Chromatogr. 2026 Aug 19;40(10):e70602. doi: 10.1002/bmc.70602 (PMC13487499; doi:10.1002/bmc.70602)
Supplement: Supplementary file 1 [file BMC-40-e70602-s001.docx]

**Investigation into the metabolism and elimination behaviour of RU 58841, and its influence on the steroid profile in humans**

A. Okutan^a^, O. Krug^a,b^, G. Fußhöller^a^, T. Piper^a^, M. Thevis^a,b^

^a^ Center for Preventive Doping Research, Institute of Biochemistry, German Sport University Cologne, Cologne, Germany

^b^ European Monitoring Center for Emerging Doping Agents, Cologne/Bonn, Germany

[Figure S1:	Product ion mass spectrum generated from the precursor ion at *m/z* 370.1373 of RU 58841 after positive electrospray ionization and collision-induced dissociation at 30 eV and proposed dissociation routes.	1](#_Toc235622034)

[Figure S2:	Product ion mass spectrum generated from the precursor ion at *m/z* 386.1322 of M1 after positive electrospray ionization and collision-induced dissociation at 30 eV and proposed dissociation routes, which were supported by pseudo‑MS³ experiments.	2](#_Toc235622035)

[Figure S3:	Product ion mass spectrum generated from the precursor ion at *m/z* 296.0652 of M2 after negative electrospray ionization and collision-induced dissociation at 30 eV and proposed dissociation routes.	3](#_Toc235622036)

[Figure S4:	Product ion mass spectrum generated from the precursor ion at *m/z* 382.1020 of M3 after negative electrospray ionization and collision-induced dissociation at 30 eV and proposed dissociation routes.	4](#_Toc235622037)

[Figure S5:	Product ion mass spectrum generated from the precursor ion at *m/z* 544.1548 of M4 after negative electrospray ionization and collision-induced dissociation at 30 eV and proposed dissociation routes.	4](#_Toc235622038)

[Figure S6:	Product ion mass spectrum generated from the precursor ion at *m/z* 558.1341 of M6 after negative electrospray ionization and collision-induced dissociation at 30 eV and proposed dissociation routes, which were supported by pseudo‑MS³ experiments.	5](#_Toc235622039)

[Figure S7:	Product ion mass spectrum generated from the precursor ion at *m/z* 560.1498 of M5 after negative electrospray ionization and collision-induced dissociation at 30 eV and proposed dissociation routes.	6](#_Toc235622040)

Figure S1:	Product ion mass spectrum generated from the precursor ion at m/z 370.1373 of RU 58841 after positive electrospray ionization and collision-induced dissociation at 30 eV and proposed dissociation routes.

Figure S2:	Product ion mass spectrum generated from the precursor ion at m/z 386.1322 of M1 after positive electrospray ionization and collision-induced dissociation at 30 eV and proposed dissociation routes, which were supported by pseudo‑MS³ experiments.

Figure S3:	Product ion mass spectrum generated from the precursor ion at m/z 296.0652 of M2 after negative electrospray ionization and collision-induced dissociation at 30 eV and proposed dissociation routes.

Figure S4:	Product ion mass spectrum generated from the precursor ion at m/z 382.1020 of M3 after negative electrospray ionization and collision-induced dissociation at 30 eV and proposed dissociation routes.

Figure S5:	Product ion mass spectrum generated from the precursor ion at m/z 544.1548 of M4 after negative electrospray ionization and collision-induced dissociation at 30 eV and proposed dissociation routes.

Figure S6:	Product ion mass spectrum generated from the precursor ion at m/z 558.1341 of M6 after negative electrospray ionization and collision-induced dissociation at 30 eV and proposed dissociation routes, which were supported by pseudo‑MS³ experiments.

Figure S7:	Product ion mass spectrum generated from the precursor ion at m/z 560.1498 of M5 after negative electrospray ionization and collision-induced dissociation at 30 eV and proposed dissociation routes.
